# Use of Intra-Operative EEG Monitoring for Nociception Balance Quantification—A Narrative Review

**DOI:** 10.3390/jcm15052072

**Published:** 2026-03-09

**Authors:** Crina-Elena Leahu, Sonia Luka, Cristina Petrisor, Sebastian Tranca, Simona Cocu, George Calin Dindelegan

**Affiliations:** 1 Department of Surgery, Discipline of Surgery, Iuliu Hatieganu University of Medicine and Pharmacy, 8 Victor Babes Street, 400012 Cluj-Napoca, Romania; 2 First Surgical Clinic, Clinical Emergency County Hospital, 3-5 Clinicilor Street, 400347 Cluj-Napoca, Romania; 3 Department of Surgery, Discipline of Emergency Medicine, Iuliu Hatieganu University of Medicine and Pharmacy, 8 Victor Babes Street, 400012 Cluj-Napoca, Romania; 4 Department of Surgery, Discipline of Anesthesia and Intensive Care, Iuliu Hatieganu University of Medicine and Pharmacy, 8 Victor Babes Street, 400012 Cluj-Napoca, Romania

**Keywords:** CONOX, qNOX, qCON, IoC1 (Index of consciousness), IoC2, nociception monitoring

## Abstract

**Introduction**: Balancing hypnosis and antinociception during general anesthesia remains challenging, as traditional clinical and hemodynamic signs incompletely reflect cortical and nociceptive processing. Electroencephalogram (EEG)-derived indices such as qCON (hypnosis) and qNOX (nociception probability) (Quantium Medical, Barcelona, Spain), as well as their predecessors IoC1 (Index of consciousness) and IoC2 (Angel-6000 A multi-parameter Anesthesia Monitor, Shenzen Weihao Kang Medical Technology Co., Ltd., Shenzen, Guangdong, China), have been developed to provide a dual assessment of anesthetic state. Their clinical role, technical limitations, and impact on drug titration, however, remain incompletely defined. **Methods**: A structured narrative review was conducted based on studies investigating IoC/qCON and qNOX in the context of anesthetic depth or nociception monitoring. Studies were grouped into three thematic domains: (1) validation against clinical or EEG standards, (2) use in guiding anesthetic or opioid administration, and (3) technical characteristics, including signal delay and pharmacodynamic modeling implications. **Results**: Sixteen studies met inclusion criteria. Eight validation studies demonstrated that IoC/qCON correlates strongly with clinical sedation scales and established EEG-derived indices such as BIS and entropy. Five interventional studies evaluating drug titration found limited impact of qCON-guided hypnosis control on anesthetic consumption but more consistent effects of qNOX/IoC2 guidance on opioid dosing and intraoperative stability. Three technical investigations showed that qCON exhibits processing delays on the order of tens of seconds that can be accounted for by incorporating monitor lag into pharmacodynamic analyses. **Conclusions**: qCON and qNOX provide complementary EEG-based indices of hypnosis and cortical nociceptive responsiveness. Evidence supports their validity as indicators of anesthetic brain state but highlights technical limitations, such as processing delay and susceptibility to physiologic factors. Their optimal clinical use lies in multimodal monitoring strategies that integrate EEG besides classic clinical and monitoring parameters.

## 1. Introduction

Personalizing the administration of anesthetic and analgetic drugs during general anesthesia, with the purpose of improving patient outcomes and safety, is a mainstay of current anesthetic practice. Besides mandatory monitoring, consisting of both clinical and vital signs, depth of anesthesia and nociception are two parameters that have also gained popularity in the last 20 years.

Nociception is the neural process by which the body detects and responds to potentially harmful stimuli [1]. Even under general anesthesia—when a person does not consciously experience pain—nociceptive signals are still generated and produce physiological stress responses. Anesthetic drugs reduce this activity, a process known as antinociception. Changes in autonomic function (such as heart rate and pupillary size) and brain activity (on EEG) have been used to assess this balance between nociception–antinociception [1,2,3].

Of the several monitors currently in use, some rely on the sympathetic–parasympathetic balance: Surgical plethysmographic index (GE Healthcare, Helsinki, Finland), Skin conductance (The Med-Storm Stress Detector, Med-Storm Innovation AS, Oslo, Norway), Analgesia nociception index (PhysioDoloris, MetroDoloris, Lille, France), Pupillometry, and Nociception level (NoL) index (Medasense Biometrics Ltd., Ramat Gan, Israel) [2,3,4,5].

There have also been systems developed based on EEG monitoring such as the qCON/qNOX monitor (Quantium Medical, Barcelona, Spain) and the Index of consciousness (IoC) monitoring (Angel-6000 A multi-parameter Anesthesia Monitor, Shenzen Weihao Kang Medical Technology Co., Ltd., Guangdong, China) [2,3,5]. EEG-derived monitoring has become central to modern anesthesia practice, aiming to reduce awareness, avoid excessively deep anesthesia, and individualize drug titration. While the bispectral index (BIS) has historically dominated this space, alternative indices such as the IoC and its successors qCON (hypnosis) and qNOX (nociception probability) have emerged.

The qCON, qNOX and IoC1 and -2 indices are derived from the frontal EEG through multistep signal processing using an Adaptive Neuro-Fuzzy Inference System (ANFIS). According to Refs. [6,7,8,9], qCON (IoC1) reflects the probability of unconsciousness or hypnotic depth and qNOX (IoC2) reflects the probability of response to noxious stimulation. The system generates an output on a scale 0–99: for qCON, a value between 40 and 60 is the recommended range during general anesthesia, with higher numbers denoting insufficient anesthetic depth; for qNOX, values within the 30–50 range reflect an appropriate level of analgesia, with values over 50 signifying inadequate analgesia [5,10].

The literature reflects the evolution of IoC/qCON from basic validation against BIS and clinical sedation scales, through technical performance analysis, and finally to perioperative outcome studies. The objective of this narrative review is to describe the uses and pitfalls of the EEG-derived parameters qNOX/qCON and IoC1/IoC2 in the current context of depth of anesthesia and nociception monitoring.

## 2. Materials and Methods

This narrative review was conducted using a structured literature search and predefined thematic categorization. Relevant publications were identified through electronic searches of PubMed, Web of Science, and Google Scholar in December 2025. The following keywords were used in various combinations: ‘qCON’, ‘qNOX’, ‘CONOX’, ‘IoC1′, ‘IoC2′, ‘depth of anesthesia’, and ‘nociception’. Following the initial electronic search, reference lists of all eligible articles were manually screened to identify additional relevant studies.

All retrieved articles were assessed individually for relevance to the scope of this review. Studies were included if they investigated IoC/qCON and/or qNOX in the context of anesthetic depth monitoring, nociception assessment, drug titration, or technical performance characteristics.

Included studies were categorized into three predefined thematic domains: validation of IoC/qCON against clinical or EEG standards, use of IoC/qCON or qNOX to guide anesthetic or opioid administration, and technical characteristics, including processing delay and pharmacodynamic modeling implications.

Data were extracted and organized into three summary tables, one for every thematic domain identified. For each study, information was categorized into six predefined fields: population, primary outcome domain, outcome, outcome measure, main result, and interpretation.

## 3. Results

The literature search identified a total of 35 studies that were narrowed down to twenty studies relevant to the use of IoC/qCON and qNOX monitoring in anesthesia, 16 of which could be grouped, based on predefined criteria, into three thematic domains. Eight studies evaluated the validation of IoC/qCON against clinical sedation scales and other established EEG-derived indices (Table 1). Five studies investigated the use of IoC/qCON or IoC2/qNOX to guide anesthetic or opioid administration and assessed their impact on drug consumption, intraoperative stability, or post-operative outcomes (Table 2). Finally, three studies examined the technical characteristics of EEG-derived indices, focusing on processing delay, responsiveness during state transitions, and implications for pharmacodynamic modeling (Table 3).

## 4. Discussion

While both indices, qCON and qNOX, are derived from the same EEG signal and therefore show some overlap, they reflect distinct but interacting components of anesthesia. In the clinical validation study by Melia et al., qCON and qNOX were compared for their ability to detect loss of consciousness (LOC) versus response to noxious stimulation. qCON predicts loss of consciousness (assessed by loss of response to verbal command and loss of eyelash reflex) better than qNOX, while qNOX gives a better reflection of the response to noxious stimulation, as assessed by lack of movement to laryngeal mask insertion [20]. During induction, qCON decreased faster than qNOX, consistent with hypnosis preceding full suppression of nociceptive responses; during recovery, qNOX increased earlier than qCON, suggesting that the return of responsiveness to painful stimuli may occur before recovery of consciousness [20]. Similarly, Jensen et al. examined the two indices during propofol–remifentanil anesthesia and validated their different physiological targets. qCON strongly correlated with BIS and reliably detected LOC, confirming its role as a hypnosis monitor [7]. In contrast, qNOX differentiated patients who moved versus those who did not during noxious stimuli (laryngoscopy, LMA insertion, and intubation) despite similar anesthetic drug concentrations, demonstrating sensitivity to nociceptive processing rather than hypnotic depth alone [7].

Although qNOX is frequently described as a “nociception” index, it does not measure nociception per se. Processed EEG indices are derived from frontal cortical electrical activity and therefore primarily reflect cortical arousal dynamics and the probability of motor responsiveness to noxious stimulation rather than the multidimensional nociceptive process itself, which encompasses peripheral transduction, spinal modulation, brainstem integration, and subcortical processing [21].

### 4.1. Factors That Influence Qcon/Qnox Values

A major theme across studies is that EEG indices do not behave identically under different anesthetic regimens. During emergence, there are different patterns of spectral EEG, according to the type of anesthetic being used, with volatiles inducing higher power in the band above 15 Hz, while propofol exhibits high power in the delta band [22]. These changes have been shown to be similarly reflected by both BIS and qCON. This indicates that index thresholds suggesting “wakefulness” may not correspond to behavioral consciousness during volatile emergence. Conversely, during propofol washout, low indices may persist despite impending responsiveness [22].

Ketamine administration revealed differential sensitivity between indices. The Patient State Index (PSI) (Sedline, Massimo, Irvine, CA, USA) increased following ketamine, whereas qCON remained comparatively stable, suggesting that nonlinear EEG metrics may be less confounded by dissociative EEG patterns [23].

Several physiological and patient-related factors have been shown to influence qCON and qNOX values beyond anesthetic type and drug concentration alone. During cardiopulmonary bypass with induced hypothermia, decreasing core temperature is associated with parallel reductions in qCON and especially qNOX, reflecting temperature-dependent slowing of cerebral electrical activity and reduced drug metabolism and clearance, which together deepen both hypnotic and antinociceptive EEG signatures [24]. Age also influences EEG-based index interpretation. Obert et al. found that both BIS and qCON tend to increase with advancing age at comparable anesthetic concentrations, likely due to age-related shifts toward relatively higher-frequency EEG activity and reduced slow-wave power under inhalatory anesthesia. Consequently, older patients may display higher index values despite adequate or even deep anesthesia [25].

### 4.2. Comparison with Other Clinical Scores and Monitors

The earliest studies establish that IoC/qCON reflects clinically observable hypnosis [8,9,26]. Revuelta showed high prediction probability between the IoC and the OAAS (Observer Assessment of Alertness/Sedation scale) during cardiac anesthesia [8]. Jensen found that IoC predicted Ramsay Sedation Scale (RSS) levels more accurately than BIS in procedural sedation [9]. Gambús further confirmed this relationship, finding that the IoC had the highest predictive probability for RSS compared with BIS and AAI/2 (autoregressive auditory evoked potential index) [26] (Table 1).

BIS has long been considered the reference processed EEG monitor for assessing hypnotic depth. Multiple comparative investigations demonstrate that qCON behaves similarly to BIS across both sedation and general anesthesia. Chakravarthy et al. [11]. found that IoC and BIS trends were similar but not numerically interchangeable, especially during non-pulsatile cardiopulmonary bypass. In Ref. [27], Müller et al. demonstrated strong correlation between qCON, BIS, and State Entropy (SE) during sedation, although qCON values were systematically lower [27]. Differences are due to scaling and algorithm design, not physiologic disagreement.

Unlike qCON, which reflects hypnosis, qNOX aims to estimate the probability of motor response to nociception. Comparison of nociception monitors highlights important physiologic differences between autonomic, brainstem, and cortical responses to noxious stimulation [12,21]. In the multimodal study by Vide et al., standardized tetanic stimuli during propofol–remifentanil anesthesia produced significant changes in pupillary reflex dilatation (PRD), analgesia nociception index (ANI), NOL, heart rate (HR), BIS, and qNOX, confirming that all indices are sensitive to acute nociceptive input. However, their relationship to opioid effect differed markedly. qNOX, derived from frontal EEG dynamics, increased after noxious stimulation and is thought to capture cortical arousal patterns associated with the probability of movement. Since it did not correlate strongly with opioid concentration, it appears to represent cortical nociceptive arousal rather than a direct measure of analgesic drug effect. These increases can probably also be traced back to alpha power suppression and increased beta activity in the raw EEG [21].

### 4.3. Using qNOX/qCON to Guide Drug Delivery (Table 2)

Use of EEG-derived indices that separate hypnosis and nociception components appears to influence analgesic drug titration rather than anesthetic drug consumption. Studies using the IoC system show that IoC1-guided hypnosis monitoring does not significantly reduce propofol requirements compared with conventional hemodynamic guidance, whereas IoC2-guided analgesia monitoring leads to higher but more precisely titrated remifentanil administration, reflecting earlier detection of nociceptive stimulation and improved intraoperative stability [14]. Similarly, during procedural sedation, IOC2 trends help identify an optimal opioid concentration that balances suppression of nociceptive responses with avoidance of cardiorespiratory depression, while higher opioid dosing correspondingly reduces propofol requirements through anesthetic–opioid synergy [15]. Evidence with the CONOX monitor follows the same pattern: qCON-guided hypnosis control does not significantly change sevoflurane consumption, but qNOX-guided analgesia results in lower fentanyl use compared with plethysmography-based guidance, suggesting more selective opioid administration [17].

As for predicting acute post-operative pain, Ledowski et al. found no association between qNOX levels at the end of the surgery, before arousal, and pain levels in the post-anesthesia care unit (PACU) [28].

### 4.4. Technical Characteristics (Delay and Modeling Implications) (Table 3)

Processed EEG-based indices are subject to computational delays that have implications for both clinical interpretation and pharmacodynamic modeling [18,19,29]. Technical evaluations using replayed EEG signals demonstrate that qCON exhibits a state-dependent time delay, with the shortest delays occurring during transitions between awake/sedation and adequate anesthesia and longer delays when entering or exiting burst suppression [29]. This behavior is comparable to other hypnotic indices, including BIS and entropy-based monitors [18,19]. These monitor delays introduce an apparent hysteresis between predicted effect-site drug concentrations and measured EEG effect. Sahinovic et al. demonstrated that by incorporating an explicit lag-time parameter (≈50–55 s for qCON), this hysteresis can be reduced, pump–monitor synchrony can be increased and estimates of effect-site concentration (Ce50) for propofol become more monitor-independent [19]. These findings underscore that qCON values should be interpreted as delayed representations of cortical state to avoid misinterpretation of responsiveness and anesthetic effect.

### 4.5. Other Clinical Uses

Using EEG-derived monitoring to guide anesthesia has been shown to decrease post-operative cognitive disfunction and stress response to surgery [10]. In a study by Qi et al., anesthesia management based on IoC monitoring led to significantly better post-operative scores for the Montreal Cognitive Assessment (MoCA) one week after surgery. This finding was linked to a lower inflammatory response in the central nervous system (CNS) as reflected by the lower levels of C-reactive protein and glial fibrillary acid protein (GFAP), a marker of neuroinflammation. Similarly, targeting moderate IoC2 ranges reduced stress hormone release and inflammatory cytokines compared with deeper or lighter anesthesia [16].

In the intensive care unit (ICU), objective EEG-derived monitoring with the CONOX system has been used as an adjunct to clinical sedation scales. qCON has demonstrated a statistically significant correlation with the Richmond Agitation Sedation Scale (RASS) in mechanically ventilated patients, supporting its feasibility for assessing sedation depth and differentiating between minimal and moderate sedation levels. A qCON threshold of approximately 80 has been associated with adequate sedation, suggesting potential utility in minimizing both under- and over-sedation [30]. The CONOX system has also been explored in deeply sedated, curarized patients in whom traditional behavioral scales such as RASS or the Behavioral Pain Scale cannot be applied. In mechanically ventilated patients receiving neuromuscular blockade for severe respiratory failure, qCON has been used to titrate hypnotic depth (target 40–60), while qNOX has demonstrated a statistically significant increase during noxious stimuli such as tracheal suction [31]. Together, these indices offer continuous, quantitative data that may enhance individualized sedation and analgesia titration in critically ill patients.

### 4.6. Limitations

This review has several limitations. As a narrative review, it does not follow a formal systematic review or meta-analytic methodology and therefore may be subject to selection bias. The included studies are heterogeneous in design, patient populations, and outcome measures, which restricts quantitative synthesis. Much of the available evidence consists of small, single-center trials focused primarily on technical validation or surrogate endpoints rather than patient-centered outcomes. The conclusions drawn should be interpreted as a structured synthesis of current evidence rather than definitive clinical recommendations.

Beyond technical validation, evidence supporting clinically meaningful outcome improvement with qCON/qNOX guidance remains limited and heterogeneous. Most interventional studies are small and primarily powered to detect differences in drug consumption or intraoperative stability rather than major patient-centered outcomes. While qNOX/IoC2 guidance appears to influence opioid titration and may improve hemodynamic control [9,10,20,22], qCON-guided hypnosis monitoring has minimal impact on volatile or propofol consumption compared with other EEG-based depth indices [6,23]. Reported benefits regarding post-operative cognitive or inflammatory outcomes are derived from exploratory or pilot studies and cannot be clearly attributed to nociception guidance alone [16]. Thus, although qCON and qNOX demonstrate physiological validity, robust multi-center trials are required to confirm reproducible improvements in hard clinical outcomes.

From a practical standpoint, the introduction of dual EEG-derived monitoring with qCON and qNOX could have implications across different procedural contexts. In total intravenous anesthesia (TIVA), where anesthetic depth cannot be deduced from end-tidal concentrations, qCON offers an objective cortical surrogate for hypnotic effect and may help reduce both unintended light anesthesia and excessive drug administration. Simultaneous qNOX monitoring can assist in detecting insufficient antinociception during surgical stimulation, particularly when hemodynamic responses are blunted by beta-blockade or vasoactive support. In volatile-based anesthesia, where MAC values already provide a population-based estimate of hypnotic depth, EEG guidance may be particularly useful during emergence in elderly patients, helping to avoid unnecessarily deep anesthesia and prolonged recovery. In short procedures and ambulatory settings, EEG-guided titration may support faster recovery profiles by minimizing anesthetic accumulation, although current evidence primarily demonstrates improved intraoperative stability rather than consistently shortened discharge times. In major surgery, especially procedures associated with significant nociceptive input, qNOX-guided opioid titration may contribute to more individualized remifentanil or fentanyl administration, potentially reducing both sympathetic overactivation and excessive opioid exposure. However, more high-quality, adequately powered prospective studies are required to determine whether these proposed clinical applications of qCON/qNOX monitoring can be implemented in a truly evidence-based manner and translate into meaningful improvements in patient outcomes.

## 5. Conclusions

Intraoperative EEG-derived indices such as qCON and qNOX represent an important step toward more objective, physiology-based assessment of anesthetic depth and nociception balance. Future research should focus on standardizing target ranges across anesthetic regimens, clarifying their role in outcome-driven protocols, and determining whether EEG-guided nociception monitoring can consistently translate into improved post-operative recovery and neurocognitive outcomes.

## Figures and Tables

**Table 1:** Validation of IoC/qCON against clinical or EEG standards.

Study	Population	Primary Outcome Domain	Outcome	Outcome Measure	Main Result	Interpretation
Revuelta 2008 [8]	Cardiac surgery under sevoflurane/remifentanil	Depth-of-anesthesia index validation	Ability of IoC to reflect clinical sedation	Prediction probability (Pk) vs. OAAS scale	IoC showed high Pk comparable to or better than BIS/CSI	IoC valid for measuring hypnotic state
Jensen 2008 [2]	110 patients undergoing deep sedation for endoscopy (propofol + remifentanil)	Hypnosis-monitoring performance Clinical sedation prediction	Agreement between IoC and BIS Ability to predict Ramsay Sedation Scale (RSS)	Correlation analysis Prediction probability (Pk)	Strong correlation between IoC and BIS IoC had significantly higher Pk than BIS	IoC tracks hypnotic depth similarly to BIS IoC better predicts sedation level than BIS
Chakravarthy 2010 [11]	Cardiac surgery patients (normotension, hypotension, CPB)	Monitor agreement	Interchangeability of IoC and BIS	Bland–Altman and correlation analyses	Significant bias and limits of agreement between monitors	IoC and BIS are not numerically interchangeable
Gambús 2011 [4]	110 patients (model development) + 68 validation patients undergoing endoscopic procedures with propofol–remifentanil TCI	Hypnosis monitoring validity Clinical sedation depth prediction	Relationship between effect-site concentrations (Ce propofol + Ce remifentanil) and EEG-based hypnosis indices Ability of EEG indices to predict Ramsay Sedation Scale (RSS)	ANFIS model performance vs. AAI/2, BIS, IoC Prediction probability (Pk)	IoC model showed best accuracy and highest predictive probability vs. BIS and AAI/2 IoC had highest Pk for predicting RSS compared with BIS and AAI/2	IoC more accurately reflects hypnotic/sedation state than BIS or AAI/2 during propofol–remifentanil sedation IoC better predicts clinical sedation level
Müller 2017 [5]	21 patients undergoing bronchoscopy with propofol sedation	Agreement between hypnosis indices	BIS vs. qCON vs. State Entropy agreement	Correlation+ agreement analysis	High correlation in trends; qCON values systematically lower	Indices behave similarly but are not numerically interchangeable
Pantalacci 2023 [12]	38 adult ASA I–III patients undergoing outpatient laparoscopic cholecystectomy	Hypnosis and nociception monitoring	Relationship between qCON and anesthetic depth Relationship between qNOX and analgesia	qCON index vs. desflurane MAC qNOX index vs. ANI	Significant negative correlation between qCON and MAC Poor but significant negative correlation	qCON reliably reflects hypnotic depth during desflurane anesthesia qNOX and ANI assess related but non-identical nociception constructs
Vide 2024 [7]	16 adults under TIVA (propofol + remifentanil) with standardized tetanic stimuli	Multimodal nociception monitoring	Response to noxious tetanic stimulation under varying remifentanil levels	PRD, ANI, NOL, HR, BIS, qNOX, raw EEG spectral changes	PRD showed strongest correlation with remifentanil concentration; ANI, NOL, and qNOX changed after stimuli but did not correlate well with opioid level	Pupillary reflex dilation may reflect opioid effect better than EEG-derived nociception indices; nociception is multimodal and not captured by a single monitor
Linassi 2024 [13]	15 adults receiving propofol–remifentanil TIVA	Relationships between EEG-derived indices	Interdependence of qCON, qNOX, EMG, and BSR, and drug concentrations	Linear modeling between indices; correlations with effect-site propofol/remifentanil concentrations	Strong linear relationship between qCON and qNOX Both strongly related to BSR at deep levels and EMG at lighter levels; qCON > 80 rarely seen without EMG activity	Processed EEG indices are not independent; EMG contamination and burst suppression strongly influence readings, limiting interpretation in deep or light anesthesia

IoC, Index of consciousness; Pk, prediction probability; OAAS, Observer Assessment of Alertness/Sedation scale; BIS, bispectral index; CSI, cerebral state index; RSS, Ramsay sedation scale; CPB, Cardiopulmonary bypass; TCI, Target-controlled infusion; ANFIS, Adaptive neuro-fuzzy inference system; AAI/2, autoregressive auditory evoked potential index; ASA, American Society of Anesthesiologists; MAC, minimum alveolar concentration; ANI, analgesia nociception index; TIVA, total intravenous anesthesia; PRD, pupillary reflex dilatation; NOL, nociception level; HR, heart rate; EEG, electroencephalogram; EMG, electromyography.

**Table 2:** Use of IoC/qCON to guide anesthetic or opioid administration.

Study	Population	Primary Outcome Domain	Outcome	Outcome Measure	Main Result	Interpretation
Wu 2016 [14]	120 patients undergoing mastectomy (IoC-guided vs. standard care)	Analgesia-guided opioid dosing	Total remifentanil dose Intraoperative adverse events	µg·kg^−1^·h^−1^ Event incidence	IoC2-guided group received significantly higher remifentanil dose IoC group had significantly fewer total adverse events	IoC2 monitoring changes intraoperative opioid titration IoC-guided analgesia improved intraoperative stability
Liu 2018 [15]	120 patients undergoing gastroscopic polypectomy	Optimal opioid dosing with IoC monitoring Optimal remifentanil concentration	Propofol requirement across remifentanil targets Balance of efficacy vs. adverse events	Total propofol dose IoC2 values + adverse events	Higher remifentanil reduced propofol dose but increased cardiorespiratory depression 4 ng/mL remifentanil provided best balance	Analgesic depth influences hypnotic requirement IoC monitoring helps identify optimal opioid dose
Zhao 2020 [16]	180 adults undergoing laparoscopic colorectal cancer resection	Optimization of anesthesia depth using IoC2	Remifentanil dose, awakening time, perioperative stress, complications	IoC2-guided groups (25–35, 35–45, 45–55) vs. remifentanil use, hemodynamics, hormones, IL-6/IL-10, QoL	Lower IoC2 (deeper anesthesia) increased remifentanil use and recovery time; higher IoC2 increased risk of intraoperative awareness; IoC2 35–45 balanced stability and had fewer complications	IoC2 appears to reflect analgesic depth; a mid-range target may optimize opioid use and perioperative physiological stability
Jehosua 2021 [6]	20 adults (ASA I–III) undergoing major laparotomy under TIVA	Anesthetic and opioid dose optimization using EEG guidance	Total propofol and fentanyl use; perioperative complications	qCON (hypnosis) and qNOX (nociception) guidance vs. standard clinical monitoring; drug consumption; hemodynamic instability episodes POCD PONV Pain in PACU	CONOX-guided group used less propofol and significantly less fentanyl Fewer hemodynamic instability episodes and lower POCD incidence No awareness in either group	EEG-guided titration with qCON/qNOX may reduce opioid exposure and hemodynamic instability and could lower risk of POCD, though findings are preliminary due to small pilot sample
Arulkumaran 2024 [17]	58 adults under sevoflurane GA randomized to AoA vs. CONOX monitoring	Anesthetic and opioid consumption	Sevoflurane and fentanyl use guided by qCON/qNOX vs. entropy/SPI	Total sevoflurane (ml/h) and fentanyl dose; recovery profile	Sevoflurane consumption similar between groups; fentanyl use lower with CONOX (qNOX-guided)	CONOX provided comparable hypnotic guidance with potentially reduced opioid administration, but qNOX interpretation depends on consciousness level

IoC, index of consciousness; µg·kg^−1^·h^−1^, micrograms per kilogram per hour; IL-6, interleukin 6; IL-10, interleukin 10; QoL, quality of life; ng/mL, nanograms per milliliter; ASA, American Association of Anesthetists; POCD, post-operative cognitive decline; PONV, post-operative nausea and vomiting; PACU, post-anesthesia care unit; GA, general anesthesia; AoA, adequacy of anesthesia; SPI, surgical pleth index; ml/h, milliliters per hour.

**Table 3:** Delay characteristics and modeling-related features.

Study	Population	Primary Outcome Domain	Outcome	Outcome Measure	Main Result	Interpretation
Kreuzer 2012 [18]	Simulated and clinical EEG recordings	Monitor technical performance	Time delay in index response to EEG state change	Seconds to reach new steady-state after EEG transitions	IoC and SE showed measurable delays (tens of seconds)	Processed EEG monitors lag behind real brain-state changes
Zanner 2020 [15]	EEG datasets + 40 patients during LOR/ROR	Monitor responsiveness Consciousness discrimination	Time delay of qCON during state transitions Ability to separate responsive vs. unresponsive states	Seconds of delay AUC	Delay ≈ 21–26 s between awake–anesthesia transitions AUC 0.63–0.90 (LOR), 0.61–0.79 (ROR)	qCON has clinically relevant processing delay qCON discriminates consciousness states moderately well to well
Sahinovic 2020 [19]	165 surgical patients (propofol–remifentanil TCI)	Monitor–drug effect synchrony PK/PD model accuracy	Delay between predicted propofol Ce and EEG index	Lag time (seconds)	Optimal lag ≈ 49 s (BIS) and 53 s (qCON) Adding lag improved model fit and produced realistic Ce50	EEG indices lag behind drug effect substantially Accounting for monitor delay improves pharmacodynamic modeling

EEG, electroencephalogram; IoC, index of consciousness; SE, state entropy; LOR, loss of responsiveness; ROR, return of responsiveness; AUC, area under the curve; TCI, target-controlled infusion; Ce, effect-site concentration; BIS, bispectral index; Pk/Pd, pharmacokinetic–pharmacodynamic.

## Data Availability

No new data were created or analyzed in this study. Data sharing is not applicable to this article.

## Abbreviations

The following abbreviations are used in this manuscript:

AAI/2	Autoregressive auditory evoked potential index
AoA	Adequacy of anesthesia
ANFIS	Adaptive neuro-fuzzy inference system
ANI	Analgesia nociception index
ASA	American Society of Anesthesiologists
AUC	Area under the curve
BIS	Bispectral index
CPB	Cardiopulmonary bypass
CSI	Cerebral state index
CNS	Central nervous system
GA	General anesthesia
GFAP	Glial fibrillary acid protein
IoC	Index of consciousness
IL-6	Interleukin 6
IL-10	Interleukin 10
EEG	Electroencephalogram
EMG	Electromyography
HR	Heart rate
LOC	Loss of consciousness
LOR	Loss of response
MAC	Minimum alveolar concentration
MoCA	Montreal Cognitive Assessment
NoL	Nociception level
OAAS	Observer Assessment of Alertness/Sedation scale
PACU	Post-anesthesia care unit
Pk	Prediction probability
Pk/Pd	Pharmacokinetic/pharmacodynamic
POCD	Post-operative cognitive decline
PONV	Post-operative nausea and vomiting
PRD	Pupillary reflex dilatation
PSI	Patient State Index
ROR	Return of response
QoL	Quality of life
RSS	Ramsay Sedation Scale
SE	State entropy
SPI	Surgical pleth index
TCI	Target-controlled infusion
TIVA	Total intravenous anesthesia
